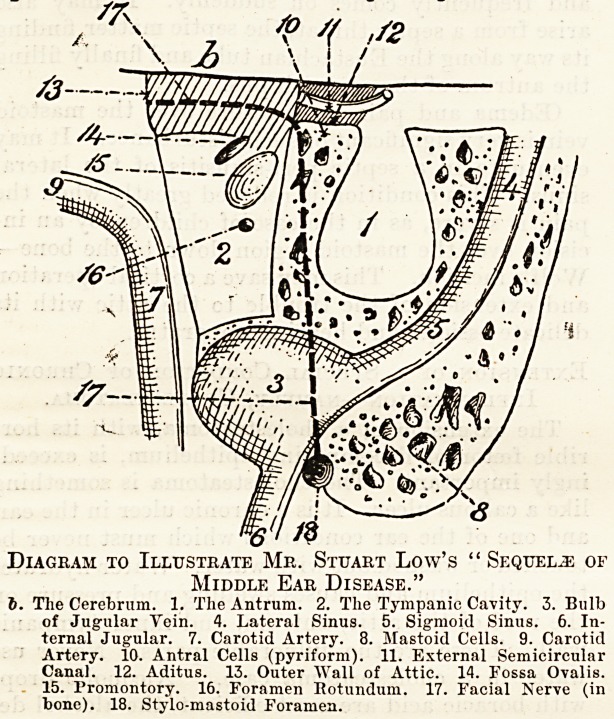# Graduate Teaching Facilities in London Hospitals

**Published:** 1906-07-28

**Authors:** 


### GRADUATE TEACHING FACILITIES IN LONDON HOSPITALS.

### CENTRAL LONDON HOSPITAL FOR DISEASES OF THE THROAT, EAR, AND NOSE.

THE graduates studying at the Central London Hospital for Diseases of the Throat and Nose in Gray's Inn Road had the advantage of a very important instruction by Mr. Stuart Low, F.R.C.S., Senior Assistant Surgeon to the hospital, as well as a teacher of practical otology at the Polyclinic. His subject is one of the utmost importance, and is of great practical interest to general practitioners, especially so to those of them who have to deal with large groups of children in public schools and academies. The subject was SOME SEQUELÆ OF SUPPURATIVE INFLAMMATION OF THE MIDDLE EAR. The following is a brief, but accurate, *résume* of the points to which he alluded, and cannot fail to be helpful:—

### EXTENSION OF SEPTIC INFLAMMATION FROM THE MIDDLE EAR TO THE BRAIN.

Mr. Stuart Low pointed out that among the SUBJECTIVE SYMPTOMS mentioned by the patient headache, due to congestion and pressure on the brain, played a prominent part. Giddiness also was important, although it might not be marked and only give rise to a slight mental confusion of ideas—the patient not being able to think well and clearly; to accomplish his mental work with the usual ease or to conserve his ideas and arrive at correct conclusions. This may only be present during some excitement causing a rush of blood to the brain, as fully described by Sir Lauder Brunton in his lectures on the Action of Medicines. The slightest incidents may bring on this confusion—getting out of bed, or suddenly assuming the erect posture, getting off a carriage, addressing an audience, or upon any sudden movement. The test of the brain is a sudden jar or exertion, just as Napoleon's test of character was emergency. Conditions like these help one greatly in diagnosis. Sickness is often found as another indication of the spread of inflammation from the mastoid cells.

The OBJECTIVE SYMPTOMS include the continuance of a profuse discharge. This may stop suddenly, and the popular notion is not altogether wrong in thinking that a sudden cessation means mischief to the brain. Examination of the ear may show with a reflected light a protrusion or bulging over the posterior part of the meatus, an indication of great importance. There may be a collection of granulations in the attic region from which pus can be sucked by means of a Siegel's pneumatic speculum. On the other hand, there may be chronic aural sepsis existing without any further symptoms, except "slow cerebration," indicative of some injury to the temporo-sphenoidal lobe and the adjoining part of the brain which controls the movements of the lips and tongue. Vomiting is the second cardinal symptom which leads to suspicion of abscess of the brain. It is well, if any doubt remains on the question of operation, to test the senses of taste and smell, for if these are markedly impaired the brain cannot be doing its work properly. In many cases the examination of the fundus oculi gives negative results, though it is helpful in meningitis. These conditions present would induce the surgeon to consider the necessity for operative interference.

Septic inflammation of the ear may be confined to the tympanic cavity, or it may extend to the mastoid cells—cells with narrow necks and wide bases. A mastoid abscess may form, indicated by increased temperature, headache, and other indications of sepsis. This condition is common after influenza, and frequently comes on suddenly. It may also arise from a septic throat, the septic matter finding its way along the Eustachian tube and finally filling the antrum of the ear with pus.

Œdema and pain on pressure over the mastoid vein is very significant of such disturbance. It may eventuate in a septic peri-sinusitis of the lateral sinus. This condition is relieved greatly when the pain is severe, as in the case of children by an incision over the mastoid region down to the bone—Wolf's incision. This may save a cortical operation and extension of the trouble to the attic with its delicate ossicles and hearing apparatus.

### EXTENSION OF A SPECIAL CONDITION OF CHRONIC INFLAMMATION—NAMELY, CHOLESTEATOMA.

The extension of a cholesteatoma, with its horrible fœtor of decomposing epithelium, is exceedingly important. The cholesteatoma is something like a callous ulcer. It is a chronic ulcer in the ear, and one of the ear conditions which must never be touched or washed out with water. Water hydrates the epithelium and causes swelling and pressure on the roof of the attic, antrum, and inner tympanic wall. These are the vulnerable parts. Never use water in a cholesteatoma case. Alcoholic drops with boracic acid are most efficient, the alcohol dehydrating and the boracic acid being slightly antiseptic.

### EXTENSION OF ACUTE OR CHRONIC INFLAMMATION TO THE LABYRINTH.

The onset of pain in the ear, a loss of the interpretation of the high-pitched sounds, and a high-pitched tinnitus afford indications that the cochlea or the labyrinth is being invaded. Pus in the labyrinth is very serious, for it is connected with the brain through the veins emptying into the inferior petrosal sinus, and more immediately by lymphatic communication.

### EXTENSION OF SUPPURATION TO THE INTRA-CRANIUM.

(1) EXTRA-DURAL ABSCESS is a common sequela, marked by great variation in the symptoms, the patient being sometimes better and sometimes worse, according to relief or increase of pressure on the brain. It is sometimes possible to suck out pus by means of Siegel's speculum, and in this way to at once relieve urgent symptoms. Nature sometimes imitates this by setting up a discharge through the roof of the attic, and the patients in this way are so much better as to refuse all operative interference.

(2) SINUS PHLEBITIS AND PYÆMIA.—Pus may be present in the groove of the lateral sinus forming a peri-sinuous abscess with pain over the mastoid region. Invasion of the lateral sinus itself may supervene, with plugging, the clot ultimately breaking down, pus entering the blood-stream, and forming pyæmic abscesses in distant parts, such as the joints and the lungs. On incision one discovers whether the lateral sinus is healthy or diseased. In health it pulsates like an artery. If pyæmia is setting in it is marked by rigors, evening and morning temperature, and other signs calling for urgent operation and removal of the clots from the sinus after tying the internal jugular.

(3) Cerebral Abscess.—Mr. Stuart Low's experience has been that the temporo-sphenoidal lobe

Diagram to Illustrate Mr. Stuart Low's "Sequelæ of Middle Ear Disease."

b. The Cerebrum. 1. The Antrum. 2. The Tympanic Cavity. 3. Bulb of Jugular Vein. 4. Lateral Sinus. 5. Sigmoid Sinus. 6. Internal Jugular. 7. Carotid Artery. 8. Mastoid Cells. 9. Carotid Artery. 10. Antral Cells (pyriform). 11. External Semicircular Canal. 12. Aditus. 13. Outer Wall of Attic. 14. Fossa Ovalis. 15. Promontory. 16. Foramen Rotundum. 17. Facial Nerve (in bone). 18. Stylo-mastoid Foramen.

is the commonest seat of abscess. Mr. Ballance,[1] on the other hand, places cerebellar abscess first. Invasion takes place through the roof of the attic and the antrum, where the bone is only one-twelfth of an inch thick, and is sometimes dehiscent. The symptoms, shortly, are slow cerebration, and pronounced vomiting with the ocular defects of external strabismus due to disturbance of the third nerve. The temperature would also be subnormal and the pulse rate reduced. If these symptoms are associated with chronic suppuration of the middle ear trephining at once is justifiable.

(4) Cerebellar Abscess.—Invasion takes place through the sinus or through the posterior and inner part of the antrum. The patient's grip is weakened on the same side as the lesion; in cerebral abscess the grip is weakened on the opposite side. This, combined with disturbance of equilibration, giddiness, vertigo, vomiting, slow cerebration to a certain extent, and a staggering gait indicate abscess in the cerebellum in a patient suffering from chronic suppuration of the middle ear, and demands operation.

Purulent Meningitis.—In meningitis the sixth nerve is implicated because of its long course, and the ophthalmoscope indicates optic neuritis. The other symptoms are fever, severe headache, vomiting, great pain, and rapid pulse. In temporo-sphenoidal abscess, in contrast with this, the pulse is slow and the temperature subnormal.

[1] "Cerebellar abscess is twice as frequent as abscess in the temporo-sphenoidal lobe." Albutt's "System of Medicine."

### Operation for Relief.

(1) The Cortical Mastoid Operation may be considered as being performed in two degrees. An incision is made down to the bone and the cells gouged away. This is the first degree of operative procedure. In doing this a little septic track or gutter, running through the bone in the cells, and sometimes leading directly into the antrum, where disease most frequently originates, is followed. The second degree consists in opening the antrum freely. This is best accomplished by gaining access from below through the mastoid cells. The antrum and surrounding parts are then freely curetted, and in this manner all granulations and carious spots in the bone are removed. The parts are then washed with antiseptic solution (biniodide of mercury, 1 in 2,000) and packed with cyanide gauze. These cases do wonderfully well; granulations spring up, and the wound heals with facility. Mr. Stuart Low emphasised the imperativeness for the more frequent performance of this most successful operation, as the disease is not only thoroughly eradicated, but the hearing is always perfectly preserved.

### Radical Mastoid Operation.

This procedure consists of throwing the tympanic cavity, the attic, the antrum, and a certain portion of the mastoid cells into one. The outer attic wall is removed and the inner, upper, and posterior part of the meatus—namely, "the bridge." It is unfortunate that the facial nerve runs through this, and that the external semicircular canal lies in the inner wall. If this canal is injured evacuation of the perilymph, and even the endolymph, may occur, and sepsis may pass backwards as far as the cerebellum. In examining the ear Mr. Stuart Low divides it into four quadrants—superior anterior, superior posterior, inferior anterior, and inferior posterior. All the important things lie in the superior posterior quadrant—promontory, foramen rotundum, fossa ovalis on the inner wall; the facial nerve runs between the fossa ovalis and the external semicircular canal down to the stylo-mastoid foramen. (See Diagram 17.) The only way to avoid the facial nerve is to open up the antrum first, and pass a probe from the antrum into the attic and chisel down outside the probe.

Mr. Stuart Low mentioned the following lines as being the best guides to operation:—

Reid's base line passes through lower margin of orbit and centre of osseous meatus (centre of the auditory meatus).

To reach the sigmoid sinus.—Base line $\frac{3}{4}$ inch behind the centre of the auditory meatus.

A cerebellar abscess is found $1\frac{1}{2}$ inch behind the centre of the auditory meatus, $\frac{1}{2}$ inch below base line.

A cerebral abscess $\frac{3}{4}$ inch above base line above the centre of the auditory meatus.

Dean's general opening is situated $1\frac{1}{4}$ inch behind the centre of the auditory meatus, and $\frac{1}{4}$ inch above the centre of the auditory meatus.

## Figures and Tables

**Figure f1:**